# Supplementary material for: Novel Chemical Architectures Based on Beta-Cyclodextrin Derivatives Covalently Attached on Polymer Spheres
Source: Polymers (Basel). 2021 Jul 16;13(14):2338. doi: 10.3390/polym13142338 (PMC8309509; doi:10.3390/polym13142338)
Supplement: Supplementary file 1 [file polymers-13-02338-s001.zip › polymers-1284898-supplementary.pdf]

Novel Chemical Architectures Based on Beta-Cyclodextrin Derivatives Covalently Attached on Polymer Spheres

Stefan Bucur[1], Ionel Mangalagiu[1,2], Aurel Diacon[3], Alexandra Mocanu[3], Florica Rizea[3], Raluca Somoghi[4], Adi Ghebaur[3,5], Aurelian Cristian Boscornea[3], Edina Rusen[3*]

[1] Faculty of Chemistry, Alexandru Ioan Cuza University of Iasi, 11 Carol 1st Bvd, 700506 Iasi, Romania

[2] Institute of Interdisciplinary Research—CERNESIM Centre, Alexandru Ioan Cuza University of Iasi, 11 Carol I, 700506 Iasi, Romania

[3]University Politehnica of Bucharest, Faculty of Applied Chemistry and Materials Science, 1- 7 Gh. Polizu Street, 011061, Bucharest, Romania

[4]National Research and Development Institute for Chemistry and Petrochemistry—ICECHIM, 202 Splaiul Independenţei, 060021, Bucharest, Romania

[5]Advanced Polymer Materials Group, University Politehnica of Bucharest, Gh. Polizu Street, Bucharest, 011061, Romania

Corresponding author: edina.rusen@upb.ro

Ts-BCD

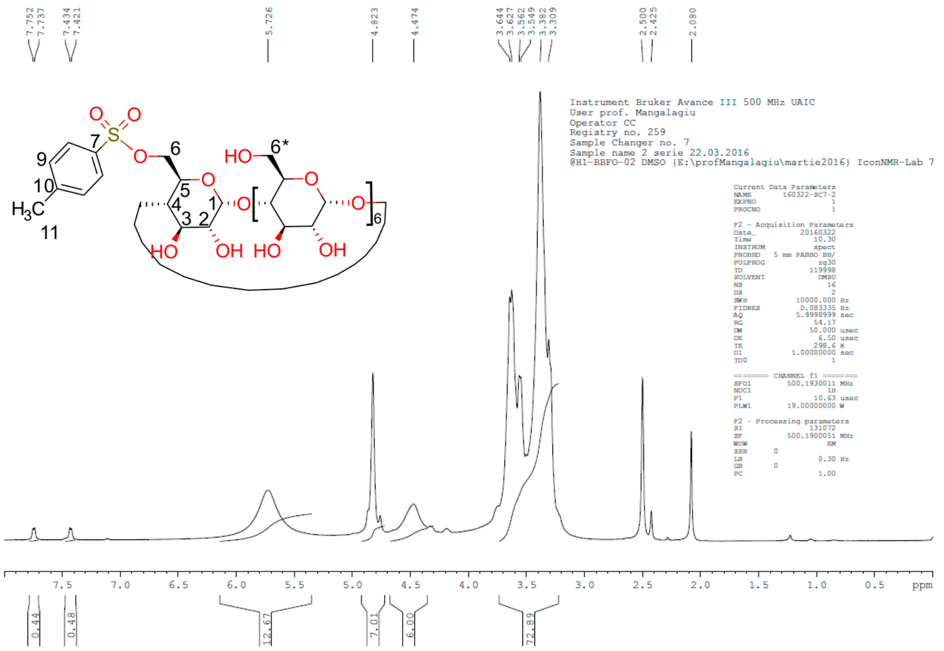

Figure S1. ^{1}H-NMR spectrum Ts-BCD

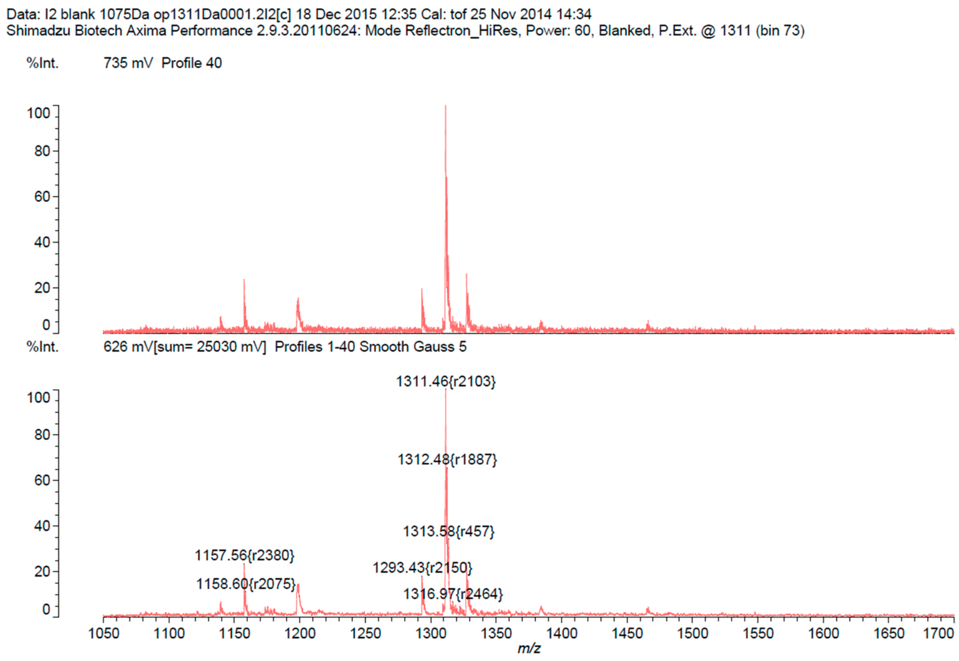

Figure S2. MALDI-TOF results for BCD-Ts

BCD-NH_2

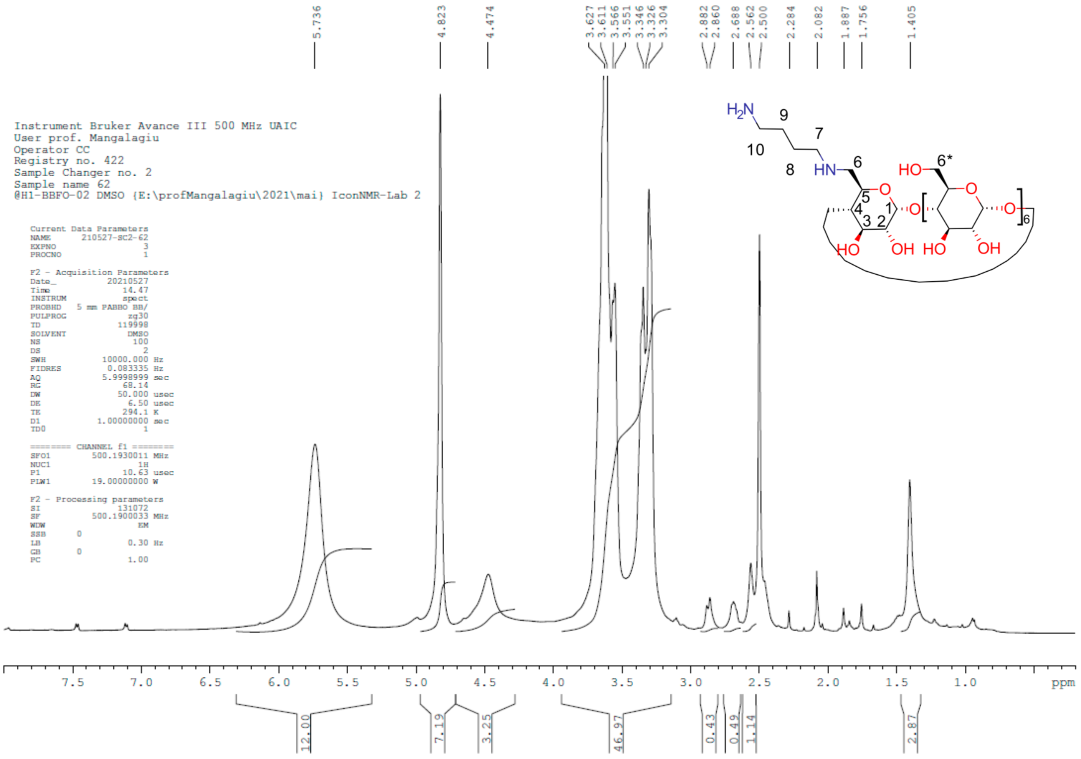

Figure S3. ^{1}H-NMR spectrum diamino butane-BCD (BCD-NH_2)

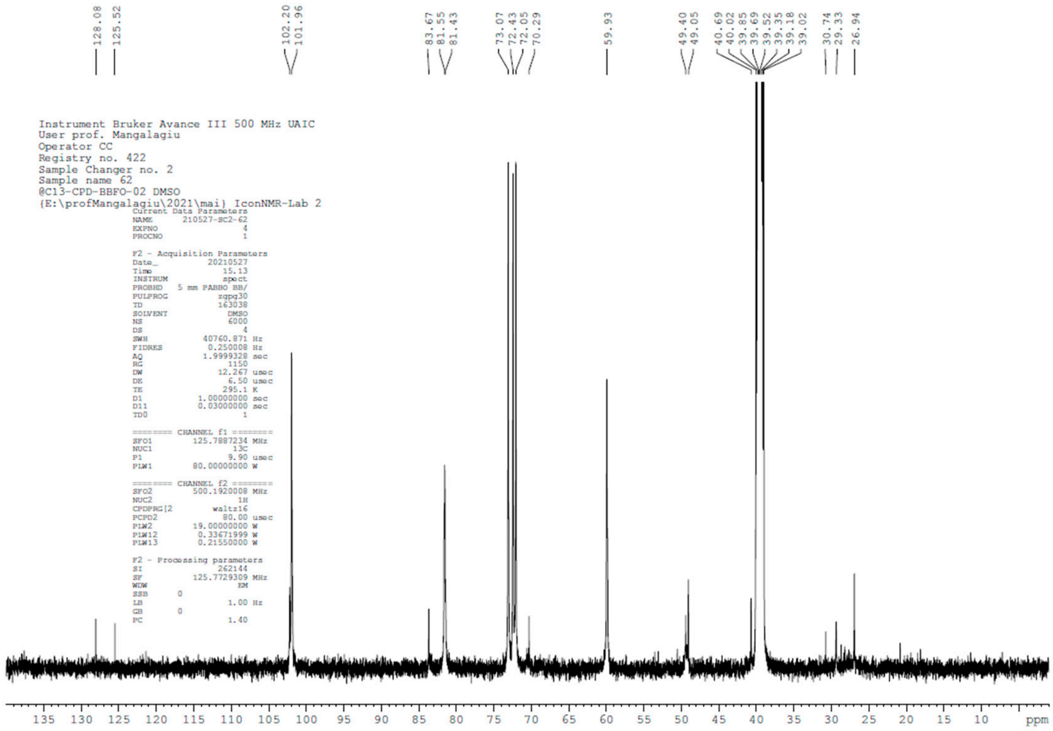

Figure S4. ^{13}C-NMR spectrum diamino butane-BCD (BCD-NH_2)

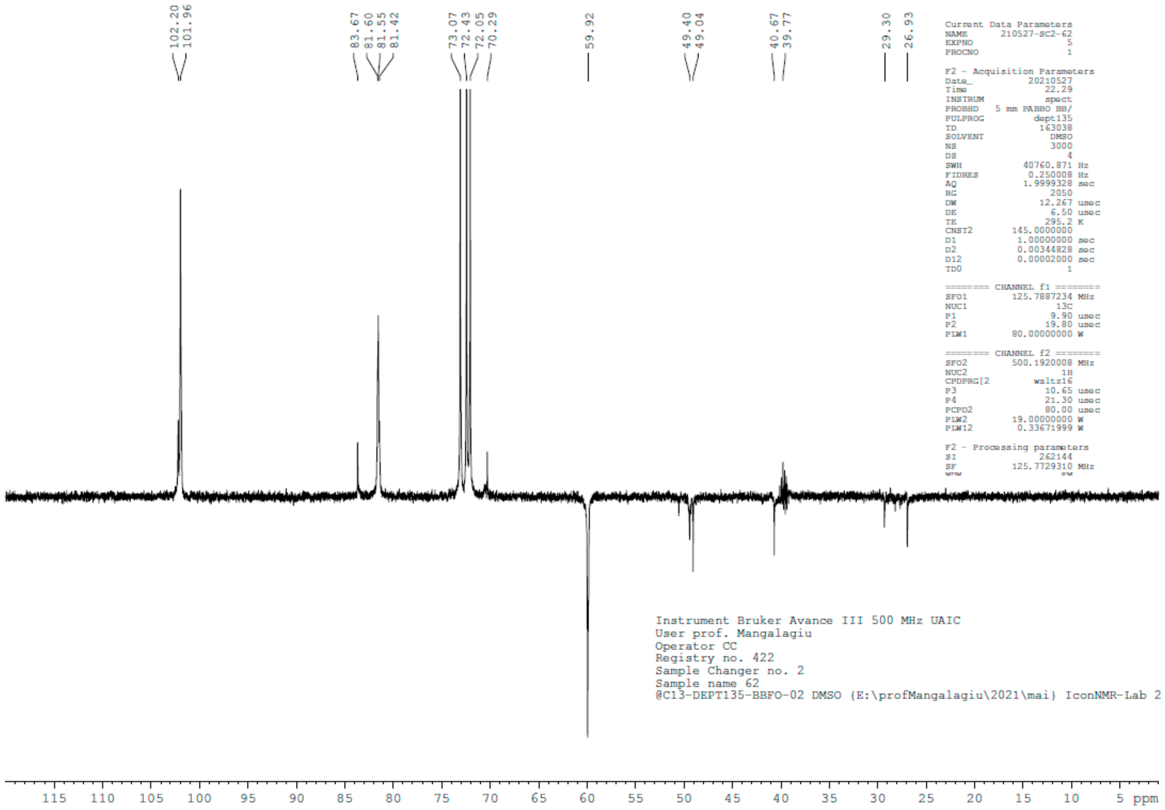

Figure S5. DEPT NMR spectrum diamino butane-BCD (BCD-NH_2)

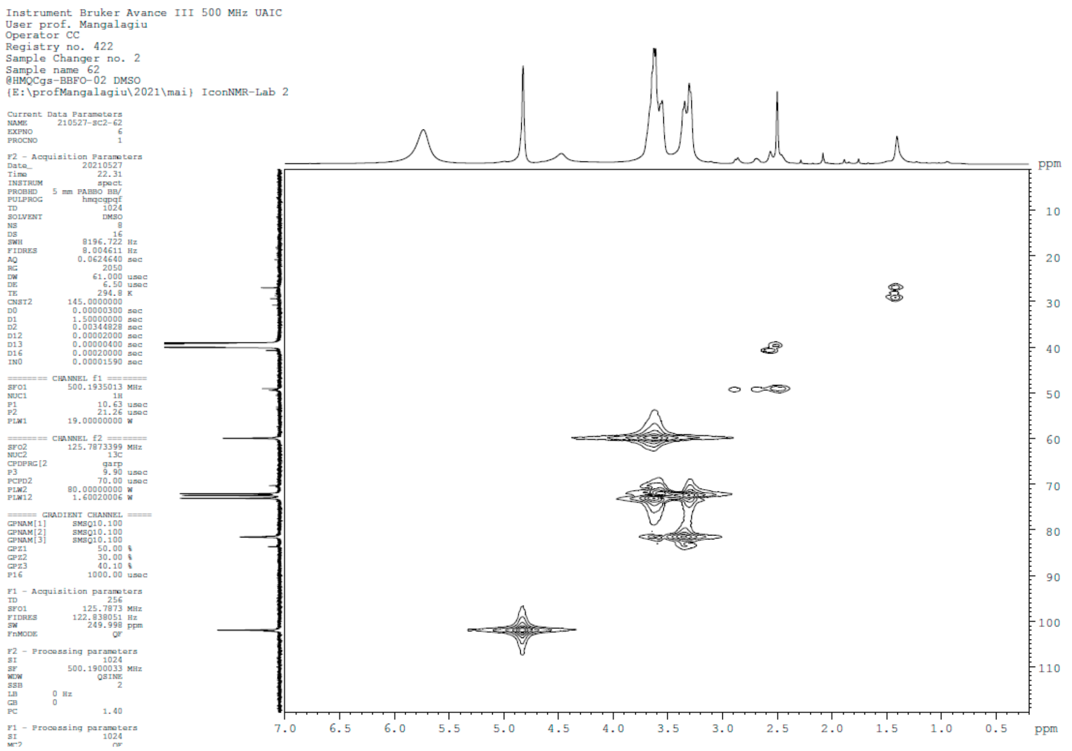

Figure S6. HMQC NMR spectra diamino butane-BCD (BCD-NH$_2$)

Determination of cyclodextrin content in the polymer

The β-cyclodextrin content was quantified by determining the reducing sugars of the polymer using concentrated H_2SO_4 acidolysis and phenol colorimetric analysis [1]. First, the method requires the preparation of a calibration curve. The solutions containing various concentrations of anhydrous glucose were mixed with 1 mL of 4% phenol standard solution and 7 mL of concentrated sulfuric acid. After vigorous shaking, the solutions were incubated at 50°C for 30 minutes and then the absorbance of the aqueous mixture was measured at room temperature. The absorbance values at 490 nm were plotted versus the concentration of anhydrous glucose to obtain the calibration curve.

The polymer analysis procedure involves the dispersing of 10 mg polymer (ST-HEMA-GMA-BCD-OH or ST-HEMA-GMA-BCD-NH$_2$) in 2 mL H_2O. After, 7 mL of concentrated sulfuric acid and 1 mL of 4% phenol standard solution were added and the solution was incubated at 80°C to ensure hydrolysis and phenol coupling reaction. The glucose level was assessed using the calibration curve and the BCD content was calculated as follows:

$$BCD\ content = \frac{c \times V \times M}{180 \times n \times 0.01} \times 100\ \%$$

Where 180 is the molecular weight of glucose, c is the glucose concentration (g/L); V is the volume of mixed solution (L); M is the molar mass of BCD (g/mol); 180 is the molecular weight of glucose, 0.01 are the grams of sample and n is the number of glucoses in a BCD unit.

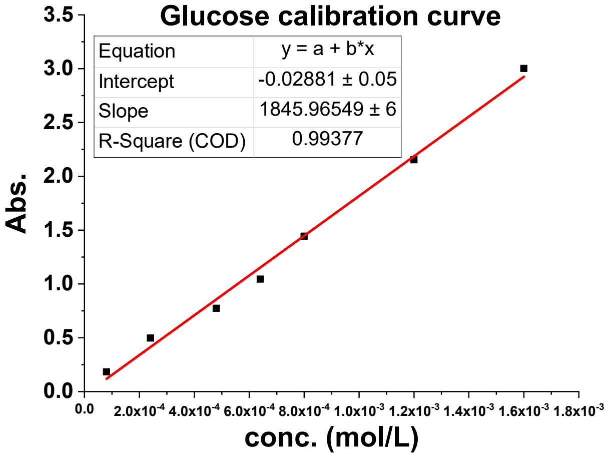

Figure S7. Glucose calibration curve (Abs. 490 nm vs glucose concentration)

Adsorption studies of bisphenol A - quantification by fluorescence spectroscopy

The adsorption of bisphenol A onto BCD substituted polymer particles ST-HEMA-GMA-BCD-OH and ST-HEMA-GMA-BCD-NH_2 was conducted using batch equilibrium technology in a mixed solution of ethanol and doubly distilled water (v:v 3:7) at the desired concentrations and a pH value of 5. In general, 0.01 g of polymer particles were dispersed thoroughly in a 50 mL solution of bisphenol A at various concentrations (5–30 mg/ L) and shaken in a thermostatic bath (shaker operated at a speed of 180 rpm at 25 °C). After equilibrium was reached, the solid sample was separated by centrifugation. The concentration of bisphenol A in the residual solution was determined using fluorescence spectrophotometry (bisphenol A: $\lambda_{ex}/\lambda_{em}$ 274/307)

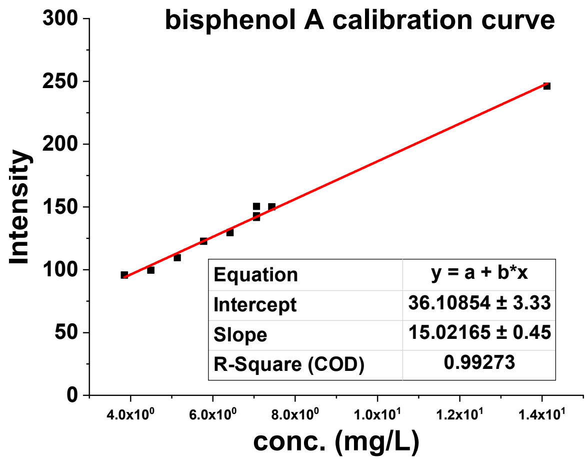

Figure S8. Plot of calibration curve for bisphenol A solution with different concentrations

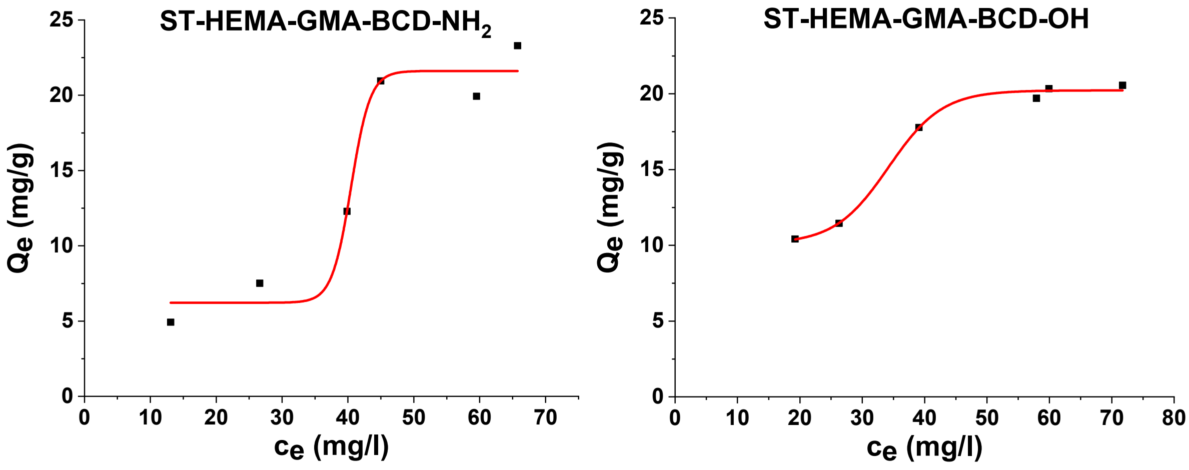

Figure S9. Equilibrium isotherms for bisphenol A at 25°C in the case of ST-HEMA-BCD-NH_2 and ST-HEMA-BCD-OH

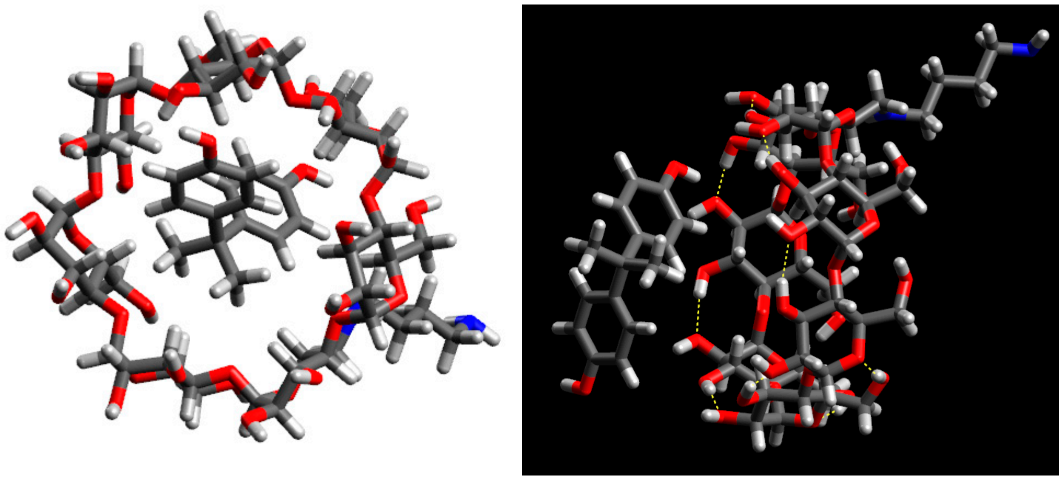

Figure S10. 3D rendering of bisphenol A insertion in BCD-NH_2

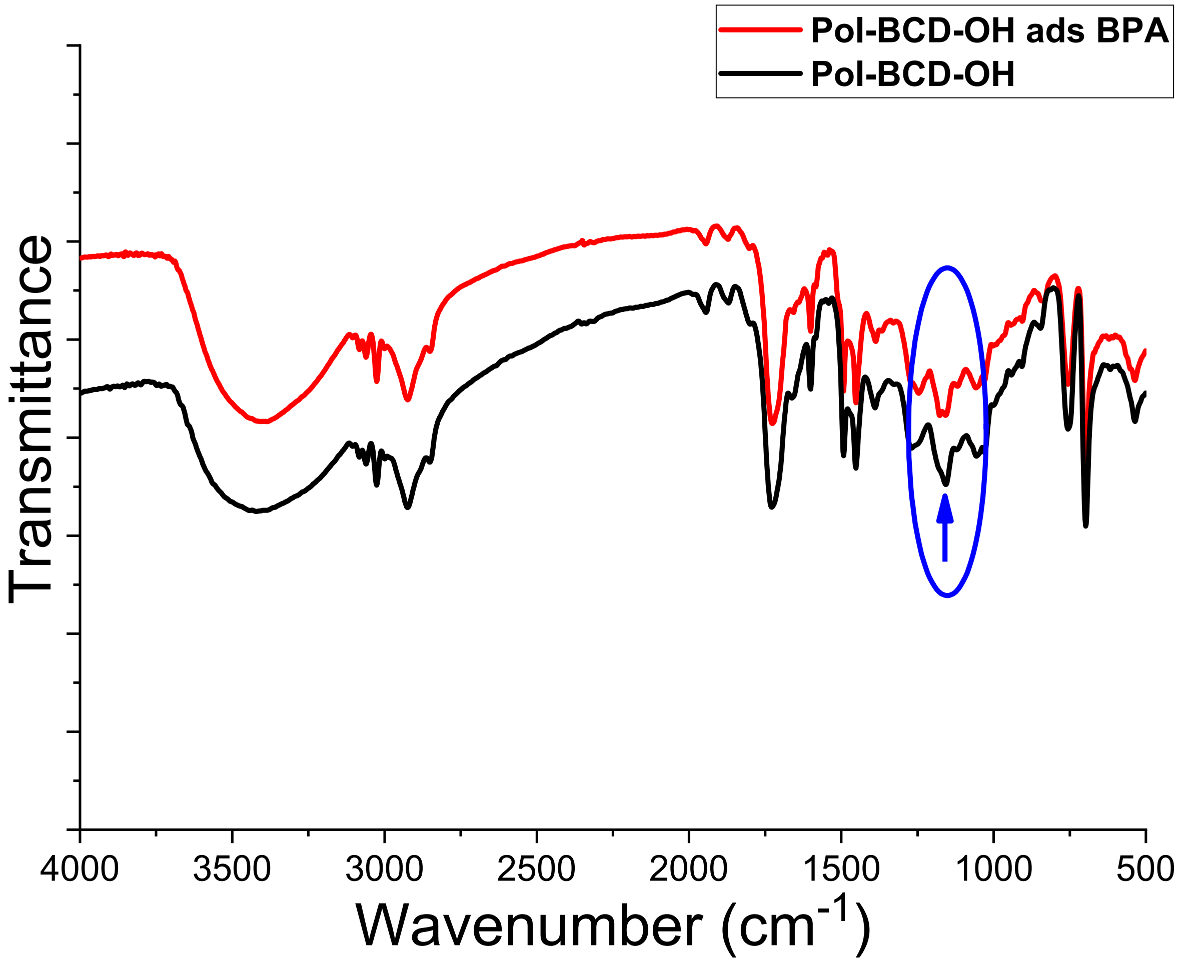

a)

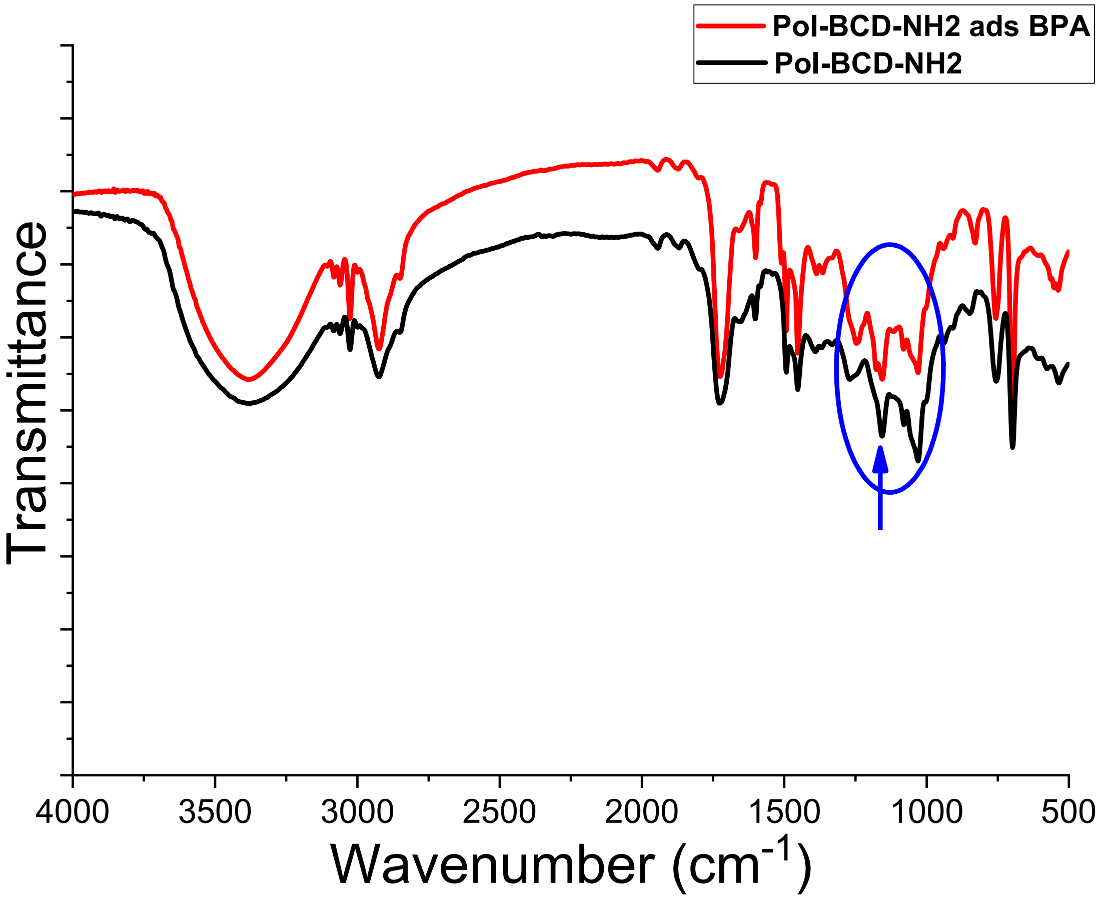

Figure S11. FT-IR spectra of the polymer materials modified with BCD derivatives after BPA adsorption study

Reference

1. DuBois, M.; Gilles, K.A.; Hamilton, J.K.; Rebers, P.A.; Smith, F. Colorimetric Method for Determination of Sugars and Related Substances. *Anal. Chem.* **1956**, *28*, 350-356, doi:10.1021/ac60111a017.
